# Mass population screening for celiac disease in children: the experience in Republic of San Marino from 1993 to 2009

**DOI:** 10.1186/1824-7288-39-67

**Published:** 2013-10-23

**Authors:** Susanna Alessandrini, Elisa Giacomoni, Fausto Muccioli

**Affiliations:** 1 Department of Pediatrics, Public Hospital of Republic of San Marino, Via Scialoja 40, 47893 San Marino, Repubblica di San Marino; 2 Central laboratory analisys, Public Hospital of Republic of San Marino, Via Scialoja 40, 47893 San Marino, Repubblica di San Marino

**Keywords:** Celiac disease, Antitransglutaminase antibodies, Screening, Children, Prevalence, Antiendomysial antibodies, Anti gliadin antibodies

## Abstract

**Background:**

Prevalence of celiac disease in developed countries is assessed about 1:100–1:150. The real prevalence is unknown because mass screenings are expensive and difficult to organize. Moreover celiac disease can affect people at every age and studies on asymptomatic subjects at different ages are not comparable. In this study we wanted to know the real prevalence of celiac disease in children in the Republic of San Marino. We also analysed concordance of different tests used and costs of mass screening.

**Methods:**

The study started in 1993. From 1993 to 1997 children aged 6, 10 and 14 were screened. Since 1997 only children aged 6 were monitored, in order to have a homogeneous population. In fact, every child born since 1980 was taken into account. Children were recruited by classroom lists of students for general paediatric examination. Until 2005 the screening test was based on dosage of antibodies anti-gliadin (AGA) IgA and IgG on venous blood. Since 2006 these tests were replaced by anti-transglutaminase IgA antibodies (ATTG). Anti-endomysial antibodies (EMA) were performed if result of any between either AGA or ATTG tests was positive or borderline; if EMA was positive, then an endoscopy with histological examination was performed to confirm the final diagnosis.

**Results:**

Attendance to paediatric examination was 96%, submission to blood test was 87%. 42 on 5092 (0,8%; 1:125) children resulted affected by celiac disease. Histology always confirmed diagnosis by serology except for two cases. AGA test (until 2005) yielded 28 on 4304 (0,7% 1:143); ATTG test (since 2006) revealed 14 positive cases on 788 (1,8%; 1:55) leading to a larger percentage of diagnosis. EMA antibodies always confirmed positivity of ATTG.

**Conclusions:**

Prevalence of celiac disease in children of Republic of San Marino is comparable to other North-European Countries. Sensitivity of ATTG proved much higher than that of anti-gliadin antibodies. Concordance between ATTG and EMA was 100%. Concordance between serology and histology was approximately 100%. Cost of screening was yearly about 5000 euros (250 children screened every year).

## 

Please see Additional file 1 for translation of the abstract into an Italian language.

## Background

The prevalence of celiac disease in occidental population is assessed about 1:100–1:150. Prevalence in children is within 1:200–1:62 when confirmed by histology and within 1:333–1:52 when assessed by serology only [1]. The real prevalence is unknown because different population screenings often use different serologic tests. Besides there are different prevalences depending on ethnic groups. In North Europe (Scandinavian Counties, Ireland and United Kingdom) some studies showed a variable prevalence within 1:100 and 1:66 [2].

Mass population screening for celiac disease has been often proposed for epidemiological studies but there are several issues to be considered. First, age of people to be screened: celiac disease can affect people at every age; then, costs and arrangement of a population screening; choice of tests; management of *potential celiacs* (subjects positive for serology and negative for histology), and finally, low compliance to gluten free diet in *silent celiacs* (subjects positive to serology and histology but asymptomatic).

Serological tests used for screening in different studies are: anti-gliadin antibodies (AGA) IgA or IgG, anti-transglutaminase antibodies (ATTG) and anti-endomysial antibodies (EMA). They have a different sensitivity and specificity.

AGA IgA have a sensitivity of 70-85% and a specificity of 70-90%. Anti-endomysial antibodies (EMA) and antitransglutaminase IgA (ATTG), together, have a sensitivity more than 95% and a specificity of about 100% [3]. Genetic test HLA DQ2/DQ8 is not a diagnostic test, but an index of predisposition to celiac disease. The classical genotype is present in about the 30% of population. It is used to exclude diagnosis in uncertain cases.

In this article we report the results of a population screening for celiac disease in scholar aged children of Republic of San Marino from 1993 to 2009.

## Methods

In Republic of San Marino mass population screening of children started in 1993. At first all children aged 6, 10 and 14 were recruited on classroom lists of schools basis. During the medical examinations, status of growth, sight, blood pressure, health of tooth and back were checked, as usual for school-aged children; in addition to this, collection of children blood sample was suggested to parents, together with explanations about celiac disease. Two paediatric health visitors planned the visits providing information to parents about the study, explaining that participation was voluntary, and collected wrote informed consent. Blood samples were taken in hospital and analysed by hospital laboratory.

Since 1997 only children aged 6 were recruited; previous calls to children aged 10 and 14 had been made in order to screen every child born from 1980 to 1990. We selected that age because in San Marino children aged 6 start full-time school, having lunch at school menses.

**Figure 1:**
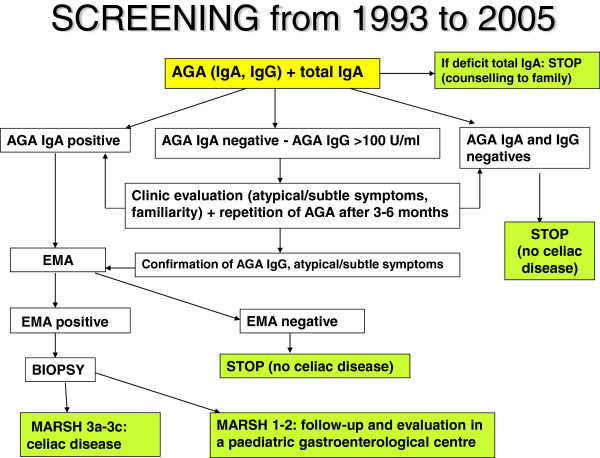
“Screening from 1993 to 2005”.

In each subject total IgA was checked, in order to exclude congenital deficit. Until 2005 we used AGA IgA e IgG (*manual ELISA test, commercial name “Celikey AGA IgG and IgA” produced by ALIFAX*) as screening test; if AGA IgA were positive (>12 U/ml), laboratory performed EMA; if AGA IgA were negative but AGA IgG were highly positive (>100 U/ml) the child was contacted again to look for subtle signs and symptoms of celiac disease (stools, abdominal pains, poor growth, loose of appetite) or familiarity for celiac disease; also, blood sample was taken again to repeat AGA and to perform EMA if indicated (*test “IFA Anti-Ema IgA assay”, produced by SCIMEDX corporation*). Thus EMA, which is an expensive test, was limited to only necessary cases, given the fact that many children had positivity of AGA IgG. If AGA IgA were negative and AGA IgG <100 U/ml, then the screening was considered negative. If EMA result was positive or borderline, an endoscopy with duodenal biopsy and histological examination were performed to assess the disease (Figure 1) “Screening from 1993 to 2005”.

**Figure 2:**
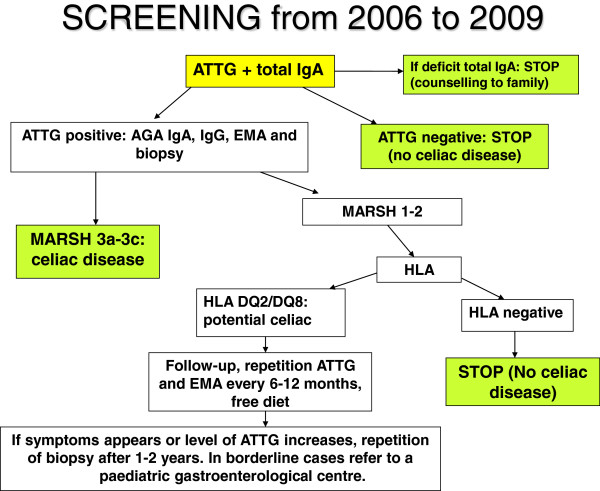
“Screening from 2006 to 2009”.

Since 2006, antibodies anti-transglutaminase IgA (*authomatic ELISA test for ALEGRIA instrument, commercial name “anti-tissue-Transglutaminase IgA”, produced by ORGENTEC*) was adopted, followed by, only if positive, EMA and AGA. An endoscopic examination with duodenal biopsy was made if ATTG were positive (> 8 U/ml) to make the final diagnosis (Figure 2) “Screening from 2006 to 2009”.

## Results

### Results of screening

Attendance to screening program was very high: on a total of 6383 children recruited, 5880 submitted to paediatric visit (96%); among them, 5092 accepted blood sample collection for the screening (87%).

42 on 5092 children resulted affected by celiac disease (positive serology confirmed by histology), with the whole prevalence referred to subjects submitted to screening (born from 1980 to 2003) of 1:125; 0,82% (confidence interval 0,8095-0,8305).

**Figure 3:**
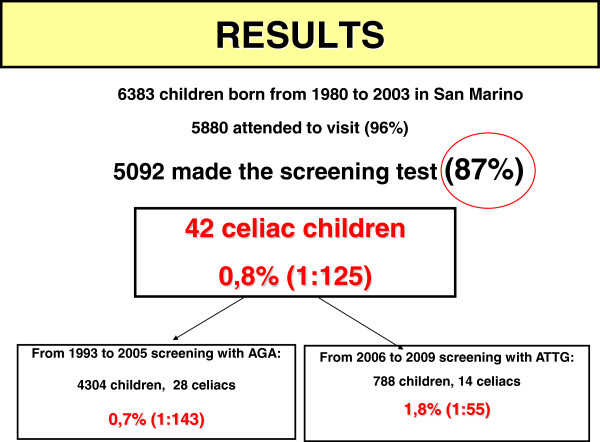
“Results of screening”.

Children screened with AGA (until 2005) were 4304 and among them we found 28 celiacs; children screened with ATTG (from 2006) were 788 and we found 14 celiacs: the percentage of celiacs diagnosed with AGA was 0,7% (1:143), while the percentage of celiacs identified with ATTG was 1,8% (1:55), about three times higher (Figure 3) “Results of screening”.

Our screening revealed 12 children with congenital IgA deficit, as expected (1:500) [4]. In those cases, we chose not to go forward with examination.

### Case-finding

Since 1993 to 2009 we found 26 celiac children out of the screening project (case-finding): in 11 children blood examination was required by paediatrics because of symptoms like abdominal pain, failure to thrive, persistent diarrhea or refractory anaemia (Hb <9 g/dl), being these children usually younger than 6 years (i.e. not included in our recruitment plan for screening). 8 children examinations were required due to at least one first degree relative affected by celiac disease (parents or brothers, these often diagnosed with population screening).

5 children resulted negative at screening time and developed the disease later. 2 of those children had AGA IgA positive at screening with negative EMA: in 1 case blood examination was repeated every year and diagnosis made 4 years later when EMA became positive; in another case parents refused to repeat blood examination every year, but when their child was admitted to our department 4 years later because of an acute abdominal pain and hypertransaminasemia, ATTG, EMA and biopsy resulted positive.

### Comparison between AGA IgA, EMA and ATTG antibodies

Since 2006, as we started using ATTG, we diagnosed 14 celiac children by screening project and 13 more by case-finding, for familiarity or suggestive symptoms. In all cases we performed ATTG, EMA, AGA IgA and AGA IgG. ATTG value was >100 U/l in 13 cases (normal < 8 U/ml), even in asymptomatic patients; ATTG was <20 U/l in 3 cases; low ATTG values often coincided with EMA borderline. All children with ATTG positive had EMA positive or borderline (index of concordance ATTG and EMA 100%).

In 17 children AGA IgA were normal (63%). In 6 children both AGA IgA and IgG were normal (22%). Symptomatic children diagnosed after 2006 with ATTG were 5; 1 had both normal AGA IgA and IgG (20%), 1 had normal AGA IgA and positive AGA IgG (20%). EMA always confirmed ATTG result.

**That means on 5 children (with or without symptoms), 1 would have not received diagnosis without performing ATTG (or EMA)** (Table 1).

**Table 1:** Comparison between ATTG, EMA, AGA IgA, AGA IgG (since year 2006)

**27 CD (14 screening + 13 case-finding) ATTG + and EMA +**		
	**TOTAL**	**SYMPTOMATIC (5 patients)**
**AGA IgA - AGA IgG +**	(17/27) 63%	(1/5) 20%
**AGA IgA - AGA IgG -**	(6/27) **22%**	(1/5) **20%**

The table shows that the 22% of total celiac patients (silent or symptomatic) and the 20% of symptomatic celiac tested since 2006 had negative AGA IgA and IgG, so they would have not received the diagnosis without performing ATTG or EMA. AGA IgG were diagnostic in only 63% of total and 20% of symptomatic celiacs.

### Overall prevalence

At the end of 2005, when screening was made with AGA, in San Marino 41 children were diagnosed to have celiac disease (28 by screening project and other 13 by case-finding) on a total of 6.943 resident children born after 1980. The prevalence of celiac disease was 1:169 or 0.59% (confidence interval 0,5784-0,6015).

At the end of 2009, in San Marino 68 patients were diagnosed for celiac disease (CD) in their childhood since 1993, 40 females and 28 males (Ratio F:M = 1,4:1). Children living in the Republic of San Marino and born after 1980 were 8.143, the overall prevalence of celiac disease (considering children diagnosed with screening and children diagnosed by case-finding) was 1:119 or 0.84% (confidence interval 0,8320-0,8480) (Table 2).

**Table 2:** Overall prevalence of CD in 2005 (AGA) and in 2009 (ATTG)

	**Population of children born after 1980**	**CD by screening**	**CD by case-finding**	**CD total**	**Prevalence**	**%**
2005 (AGA test)	6.943	28	13	41	1:169	**0,59%**
+ 4 years	+ 1.200	+ 14	+ 13	+ 27		
2009 (ATTG test)	8.143	42	26	68	1:119	**0,84%**

The table shows the increasing of diagnosis of celiac disease between children (from 0,59% to 0,84%) after introduction of ATTG (considering children diagnosed by screening and by case-finding in 4 years).

### Histological diagnosis was uncertain in 4 cases (2 in screening and 2 by case-finding)

1) A female aged 6, affected by Turner syndrome, asymptomatic, submitted to screening. AGA and EMA antibodies were positive, but histology completely negative. We performed fraction gamma-delta T cell receptor, that resulted negative; the HLA was DQ2DQ8; her father was affected by celiac disease.

2) A male, little-brother of the girl reported above, with normal karyotype but affected by mental retardation and multiple malformations (vesicoureteral reflux, strabismus, cryptorchidism). When, at the age of 2, the child was visited for recurrent abdominal pains, AGA and EMA resulted positive. Histology resulted negative and so was the examination of fraction gamma-delta T cell receptor. The HLA was DQ2DQ8. For that patient and his sister a paediatric gastroenterological centre suggested a gluten-free diet, which was started with benefit;

3) A female, aged 6, at first asymptomatic, with borderline serology at the screening (ATTG 33 U/l, EMA borderline), histology MARSH 1 and HLA DQ2; we let her at free diet, but some weeks later she had persisting diarrhea and hyperactivity; during this period her father was diagnosed for celiac disease (with intestinal atrophy). After starting gluten free diet (suggested by a paediatric gastroenterological centre), diarrhea and hyperactivity resolved;

4) A female, aged 6, visited out of our screening plan for alternating constipation and diarrhea, hyposomia and recurrent abdominal pain. Serology was highly positive (ATTG > 100 U/l, EMA positive) but histology showed a MARSH 1; also in this case, the gluten-free diet resolved symptoms.

## Discussion

As a consequence of the presented screening program it could be said that “the population screening amplified the screening itself”. In fact, identification of celiac patients through screening program lead to check of many asymptomatic subjects simply because of their relationship with each other. Moreover public awareness about celiac disease grew up: an association of celiac patients was founded, more information was available to population and family doctors, restaurants started offering special menus for celiacs, school lunches for celiacs became mandatory. Doctors with increased awareness towards the disease made it possible to raise number of diagnosis out of screening program.

The attendance to screening, given all the facts mentioned above, was very high compared to similar studies in Italy [5]. We had a great advantage in being a little Republic: just seven paediatricians (for the whole population aged 0–14) working together in the same hospital and in strict collaboration with schools. In practice, screening project took place during already scheduled ordinary visits and parents were provided with information by doctors they trusted in, that made parents and children feel comfortable. Laboratory analysis were committed locally at the hospital, thus minimizing cost and handling.

For what concerns analysis results, ATTG antibodies showed to be about three times more sensitive than anti gliadin in identifying celiac patients, and the possibility to confirm the diagnosys at this stage is under discussion, as suggested by some guidelines [6,7], since EMA always confirmed results of ATTG and is an expensive test.

### Limitations of the screening program

In our screening project total IgA antibodies of all patients were measured to exclude a deficiency. In case of IgA deficiency in asymptomatic children without familial risk for the disease, it was necessary to minimize invasive procedures. Consequently, parents were informed about their children’s benign immunological condition and not diagnostic result of the screening for celiac disease, together with the advise to perform further examinations only if the child would show symptoms or if there was a first-degree relative affected. Until now, we have not reported any case of celiac patient with IgA deficiency.

Diagnosis in patients with a positive serology and a normal or not diagnostic histology (MARSH 1–2) is an issue of major concern: those patients must be checked for the genetic susceptibility HLA. If HLA results predisposing to disease (*potential celiac*) a long-term follow-up is required with repetition of ATTG every 12 months and further endoscopies. At the moment there are different guidelines for the follow-up of potential celiacs: some authors suggest a challenge with large quantity of gluten before repetition of biopsy, others suggest to perform the mucosal ATTG. A recent study showed that potential celiac children followed-up for 3 years developed mucosal atrophy in one-third of cases [8]. In those cases, a long term follow-up is mandatory for stating the correct diagnosis.

Speaking of the compliance to gluten-free diet in asymptomatic subjects (*silent celiacs*), a recent study demonstrated that compliance is good within children after population screening [9] and that applied to our patients, too. In adolescence the compliance to diet decreases, because teenagers have often meals out of home. In our experience, the mass population screening increased awareness about celiac disease and that gave young patients the opportunity of understand the importance of diet.

#### Costs of population screening

We considered an approximate cost of € 15 for ATTG antibodies, € 25 for EMA antibodies, € 5 for total IgA antibodies, € 11,40 for AGA IgG plus IgA and € 300 for HLA.

In our population, performing ATTG antibodies and total IgA on an average of 250 children every year costs yearly about € 5000 which grows up to 11250 euros if EMA is considered for every child.

To take into account HLA costs, HLA examination is performed once-in-life for first-degree relatives of celiac patients: as every year we diagnosed an average of 4 children (since 2006 with ATTG) and considering 3 first-degree relatives for each one (parents and one brother or sister), the cost of HLA analysis is about € 3600 yearly.

In addition there are costs of endoscopies (€ 200/each). We did not consider costs for medical visits and laboratory workers because the screening program was carried out during ordinary paediatric visits and serological testing were performed by hospital laboratory during normal activity. We also didn’t consider costs for gluten-free diet (Table 3).

**Table 3:** Costs of screening

	**ATTG (€ 15) + total IgA (€ 5)**	**EMA (€ 25) + total IgA (€ 5)**	**Endoscopies**	**HLA**
Each	€ 20	€ 30	€ 200	€ 300
For 1000 subjects			*Only if positive serology (about 1:100 screened)*	*Only in CD 1° degree relatives (4 patients)*
	€ 20000	€ 30000	€ 2000	€ 12000

While costs of population screening are easy to calculate, benefits are not. There are no studies comparing the health of asymptomatic celiac subjects maintaining a free diet, towards asymptomatic celiac subjects observing an early gluten-free diet. A recent study on 32 asymptomatic children diagnosed for celiac disease (thanks to a screening between 2 and 4 years) showed that after 10 years 66% of children did not show a deterioration of generic health-related quality of life. The authors concluded that “long-term follow-up studies are needed to assess possible long-term complications in untreated, non-symptomatic celiac disease” [9].

#### Future perspectives

Some authors proposed not to perform endoscopy and biopsy on classic symptomatic patients (diarrhea, failure to thrive, malabsorption) with high levels of ATTG (>100 U/l) [6]. Those patients after some months of a gluten-free diet have the resolution of symptoms and normalization of serology [10]. In our experience, when ATTG antibodies were higher than 100 U/l, histology always showed a mucosal atrophy (MARSH 3a-3c), except for one case.

The biopsy in children is an invasive exam, even if always well-tolerated, and often requires sedation. Some authors propose to postpone biopsy in symptomatic children with highly positive ATTG, to start a gluten-free diet for some months and to make the biopsy only if symptoms persist. Currently, population screening needs biopsy to make the definitive diagnosis, because subject are asymptomatic. We suggest to postpone the biopsy when serology values are borderline (ATTG < 3 times normal), follow up the child at a free diet, repeating ATTG every 6–12 months and evaluating the appearance of symptoms. Some cases of spontaneous normalization of serology are described in literature, as we also experienced.

## Conclusions

The screening program brought to an increased awareness of doctors and education of people about celiac disease. “The screening program amplified itself” because often non-symptomatic relatives of children affected were investigated and diagnosed for celiac disease.

ATTG is the gold standard for screening, it has the same sensibility of EMA and it is less expensive: we found a concordance between ATTG and EMA of 100%. When ATTG values were less than 3 times normal (<20 U/l), EMA were borderline. In our experience, 1 child on 5 (20%) even if symptomatic, would have not received the diagnosis if only AGA IgA and IgG tests were used. Histology always confirmed ATTG and EMA test, except for 4 cases, which were diagnosed few years later by means of follow-up.

General prevalence of celiac disease in Republic of San Marino is comparable to other countries of North Europe (about 1:100); nonetheless the value tends to increase, as ATTG (used since 2006) reveals having found about 4 non-symptomatic celiac 6-years-old children on 250 tested every year, we can state that in some years the general prevalence will be next to 1:50.

However we can not conclude that there is an increasing of diagnosis of celiac disease, because ATTG was introduced in 2006 and population screened from 2006 to 2010 is not relevant, yet.

In the end, the screening is not too expensive: about 5000 euros for 250 children yearly for ATTG and total IgA (about 20 euros per child). If included in ordinary paediatric visits it’s easy to promote and compliance is good.

Issues still open are the diagnosis and follow-up of non-symptomatic patients with a positive serology and a normal or not diagnostic histology (MARSH 1–2) and management of subjects with IgA deficiency. In a future perspective a non-invasive test replacing biopsy for a definitive diagnosis in non-symptomatic children is auspicable.

## Abbreviations

ATTG: Antitransglutaminase antibodies; EMA: Antiendomysial antibodies; AGA: Anti gliadin antibodies; IgG: Immunoglobulin G; IgM: Immunoglobulin M; CD: Celiac disease.

## Competing interests

The authors declare that they have no competing interests.

## Authors’ contributions

AS conceived the study, participated in its design and coordination. GE performed analysis of data and draft the manuscript. MF performed EMA tests. All authors read and approved the final manuscript.

## Authors’ information

Dr. A. S. is a paediatrician working in Public Hospital of San Marino. She has always been interested in paediatric gastroenterology, and is the contact doctor for the Association of Celiac Patients in San Marino. She is a member of Regional Group of Paediatric Gastroenterology.

Dr. G. E. is a young paediatrician working in Public Hospital of San Marino.

Dr. M. F. is a Biologist working in Central Laboratory Analisys in Public Hospital of San Marino.

## Supplementary Material

Additional file 1 Abstract in Italian language. Click here for file
