# Supplementary material for: Mass population screening for celiac disease in children: the experience in Republic of San Marino from 1993 to 2009
Source: Ital J Pediatr. 2013 Oct 23;39:67. doi: 10.1186/1824-7288-39-67 (PMC3875895; doi:10.1186/1824-7288-39-67)
Supplement: Additional file 1 — Abstract in Italian language. [file 1824-7288-39-67-S1.pdf]

Abstract

Background

La prevalenza di malattia celiaca nei paesi sviluppati è stimata intorno a 1:100–1:150. La prevalenza reale non è nota perché gli screening di massa sono costosi e pongono difficoltà organizzative. Inoltre la celiachia può presentarsi ad ogni età, quindi gli studi su soggetti asintomatici di età diverse non sono confrontabili. L'obiettivo di questo studio è conoscere la prevalenza reale di celiachia nei bambini della Repubblica di San Marino. Inoltre abbiamo analizzato la concordanza tra i vari test sierologici e considerato i costi dello screening.

Materiali e metodi

Lo studio è iniziato nel 1993; fino al 1997 abbiamo reclutato bambini di 6, 10 e 14 anni, poi solo bambini di 6 anni per avere una popolazione omogenea. In questo modo sono stati chiamati tutti i bambini nati dal 1980 in poi, utilizzando le liste di iscrizione scolastica. I bambini venivano invitati per una visita pediatrica di base, durante la quale il pediatra spiegava cosa fosse la celiachia e proponeva ai genitori di fare un prelievo venoso. Fino al 2005 lo screening si è basato sul dosaggio degli anticorpi anti-gliadina (AGA) IgA e IgG, dal 2006 sul dosaggio delle anti-transglutaminasi IgA (ATTG). Gli anticorpi anti-endomisio (EMA) venivano eseguiti solo se AGA o ATTG risultavano positivi o borderline, se gli EMA risultavano positivi veniva eseguita la biopsia duodenale.

Risultati

la partecipazione allo screening è stata dell'87%, per un totale di 5092 bambini, di cui 42 sono risultati affetti (0,8%). La diagnosi istologica ha confermato la positività sierologica in tutti i casi tranne 2. I bambini sottoposti a dosaggio degli anticorpi antigliadina fino al 2005 sono stati 4304 con 28 celiaci (0,7% 1:143); dal 2006 i bambini sottoposti ad ATTG sono stati 788 con 14 celiaci (1,8% 1:55). Gli EMA hanno sempre confermato la positività degli ATTG.

Conclusioni

La prevalenza della celiachia nei bambini di San Marino è simile a quella dei paesi del Nord Europa. Gli ATTG sono risultati 3 volte più sensibili rispetto agli anticorpi anti-gliadina. La concordanza tra ATTG ed EMA è risultata del 100% e tra sierologia e biopsia è risultata approssimativamente del 100%. Il costo stimato dello screening è circa 5000 euro per 250 bambini all'anno.
